# Beyond the Norm: A Unique Case of Adult-Onset Still’s Disease

**DOI:** 10.7759/cureus.68104

**Published:** 2024-08-29

**Authors:** Bahaa Attia, Mohammed Said Ismail, Nehal El-Ghobashy, Hala Farawela, Kamal EL Garf, Mohamed Abdelkader Morad, Megha Lokesh

**Affiliations:** 1 Critical Care Medicine, King's College Hospital NHS Foundation Trust, London, GBR; 2 Pulmonary Medicine, Cairo University, Kasr Alainy Hospitals, Cairo, EGY; 3 Rheumatology and Rehabilitation, Cairo University, Kasr Alainy Hospitals, Cairo, EGY; 4 Clinical and Chemical Pathology, Cairo University, Kasr Alainy Hospitals, Cairo, EGY; 5 Internal Medicine and Clinical Hematology, Cairo University, Kasr Alainy Hospitals, Cairo, EGY; 6 General Medicine, King's College Hospital NHS Foundation Trust, London, GBR

**Keywords:** tociluzimab, elderly onset stills disease, bilateral pleural effusion, adult-onset still’s disease, high fever

## Abstract

Polyserositis, characterized by inflammation of multiple serous membranes, frequently occurs secondary to infection, malignancy, or rheumatological disorders. Adult-onset Still's disease (AOSD) is often diagnosed by exclusion, with the Yamaguchi criteria being essential for diagnosis. Disease severity is likely due to immune system changes, comorbidities, delayed diagnosis, and a higher risk of complications, necessitating more aggressive and carefully monitored treatments. We report the case of an elderly male who was diagnosed with AOSD by exclusion using the Yamaguchi criteria. The patient presented with bilateral pleural effusion, systemic inflammation, arthralgia, and fever. Initial investigations included complete blood count, C-reactive protein, and erythrocyte sedimentation rate, which revealed a severe acute phase reactant. Imaging studies, including chest X-ray and CT scan, revealed bilateral pleural effusion. Despite traditional treatment approaches, such as high doses of steroids and other immunosuppression medications, the patient's condition remained refractory, indicating the complex and challenging nature of managing AOSD in elderly patients. The increased severity and higher complication rates in older individuals require a multidisciplinary approach to ensure optimal outcomes. Aggressive treatment strategies, vigilant monitoring, and thorough diagnostic workups are essential to manage the disease effectively. This case highlights the need for heightened awareness and consideration of elderly onset Still's disease (EOSD) in differential diagnoses for elderly patients presenting with polyserositis and systemic inflammatory symptoms.

## Introduction

Adult-onset Still's disease (AOSD) indeed presents diagnostic challenges due to its nonspecific clinical features and lack of clear diagnostic tools. Typically presents with initial features including a sore throat, fever above 39°C for more than a week, arthralgia, a salmon-colored rash, hepatosplenomegaly, lymphadenopathy, leucocytosis with polymorphonuclear neutrophils (PMNs) over 80%, elevated liver enzymes, and other inflammatory markers.

The pathogenesis of which also is not completely understood, although generally believed to involve a complex interplay between dysregulated innate and adaptive immune responses, leading to excessive cytokine production and systemic inflammation. Understanding these mechanisms is crucial for developing targeted therapies to manage this challenging condition. There is also thought to be both an infectious and genetic element involved in its development [1].

Internal organ inflammation is a less common side effect of ASOD. Pleuritis, or inflammation of the lung membrane, can also occur in certain patients and result in pleural effusions, or the accumulation of fluid around the lungs. Effusions in AOSD are typically exudative, indicating a high protein content and inflammatory nature. The treatment of effusions in AOSD involves managing the underlying inflammatory process with medications such as corticosteroids, nonsteroidal anti-inflammatory drugs (NSAIDs), and biologics targeting specific cytokines (e.g., interleukin-1 or interleukin-6 antagonists) [2].

The understanding of adult-onset Still's disease (AOSD) in the advanced age group remains insufficient due to several factors, including the lack of well-defined clinical features, the absence of specific diagnostic tools, and the often aggressive nature of the disease [3]. 

In this case, a comprehensive differential diagnosis was conducted to accurately identify adult-onset Still's disease (AOSD). Potential conditions with overlapping symptoms such as fever, rash, arthritis, and systemic inflammation were carefully considered and systematically ruled out. This included infectious causes (bacterial, viral, tuberculosis, fungal), rheumatic diseases (rheumatoid arthritis, systemic lupus erythematosus (SLE), vasculitis), malignancies (lymphomas, leukemias, solid tumors), autoinflammatory or autoimmune disorders, and other systemic inflammatory conditions (sarcoidosis, inflammatory bowel disease). Diagnostic tools, including laboratory tests, imaging studies, and biopsies, were employed to exclude these conditions, confirming the diagnosis of AOSD based on clinical criteria and response to treatment.

## Case presentation

A 72-year-old male patient with a past medical history of well-controlled bronchial asthma and dyslipidemia presented with acute onset of severe sore throat, myalgia, arthralgia, and fever spikes >40c. Febrile episodes persisted despite the prolonged course of broad-spectrum antibiotics; the episodes were of a rhythmic pattern; a high temperature was preceded by episodes of shivering and lasted for 36 hours despite cooling measures. A pink purpuric rash was noted associated with the febrile episodes, although it was initially difficult to distinguish given the patient's darker skin tone.

Initial blood test revealed elevated white cell count 20.5 x 10^3^/cm with neutrophilic predominance of 92%, C-reactive protein (CRP) 394 mg/L, erythrocyte sedimentation rate (ESR) 15 mm, ferritin 647 & 538 ng/ml, respectively, lactate dehydrogenase (LDH) 360 U/L, albumin 2.1 g/dl, aspartate aminotransferase 386 U/L, and alanine aminotransferase 125 U/L. Septic screen was noted to be negative, which included sputum, urine, and blood culture along with COVID and respiratory screen. Extensive blood workup performed with negative results in the form of Mycobacterium tuberculosis from different sites (sputum and pleural fluid), cytomegalovirus (CMV), Epstein-Barr virus (EBV), herpes simplex virus (HSV), malaria, Brucella, yellow fever, dengue fever, hepatitis C virus (HCV), hepatitis B virus (HBV), human immunodeficiency virus (HIV), flow cytometry, urine & serum electrophoresis. Cerebrospinal fluid analysis showed a normal cell count with no evidence of infection. Thyroid function tests and cortisol levels returned normal. Autoimmune profile in the form of antinuclear antibodies (ANA), anti-DNA antibodies, C3, C4 extractable nuclear antigen, rheumatoid factor (RF), anti-cyclic citrullinated peptide (CCP), and anti-neutrophil cytoplasmic antibodies (ANCA P & ANCA c) within normal value. IL-6 (interleukin-6) 256 Pg/ml was markedly elevated (Table 1).

**Table 1:** Blood test result and reference.

Serial	Blood test	Result	Unit	Normal reference
1	White cell count (WCC)	20.5x10^3^	cmm	4-11
2	Neutrophil	92	%	40-75
3	Aspartate transaminase (AST)	386	U/L	Up to 50
4	Alanine transaminase (ALT)	125	U/L	Up to 50
5	Albumin (Alb)	2.1	gm/dL	3.2 – 4.6
6	Erythrocyte sedimentation rate (ESR)	15	mm	Up to 30
7	Ferritin	647	ng/mL	22 – 322
8	Lactate dehydrogenase	360	U/L	0 – 247
9	Creatinine	1.8	mg/dL	0.8 – 1.43
10	C-reactive protein (CRP)	394	mg/L	Up to 5
11	Interleukin-6 (IL-6)	256	Pg/mL	Less than 7
12	Blood culture	No growth	-	-
13	Sputum culture	No growth	-	-
14	Urine culture	No growth	-	-
15	Covid-19 polymerase chain reaction (PCR) & respiratory panel	Negative	-	-

**Figure 1:**
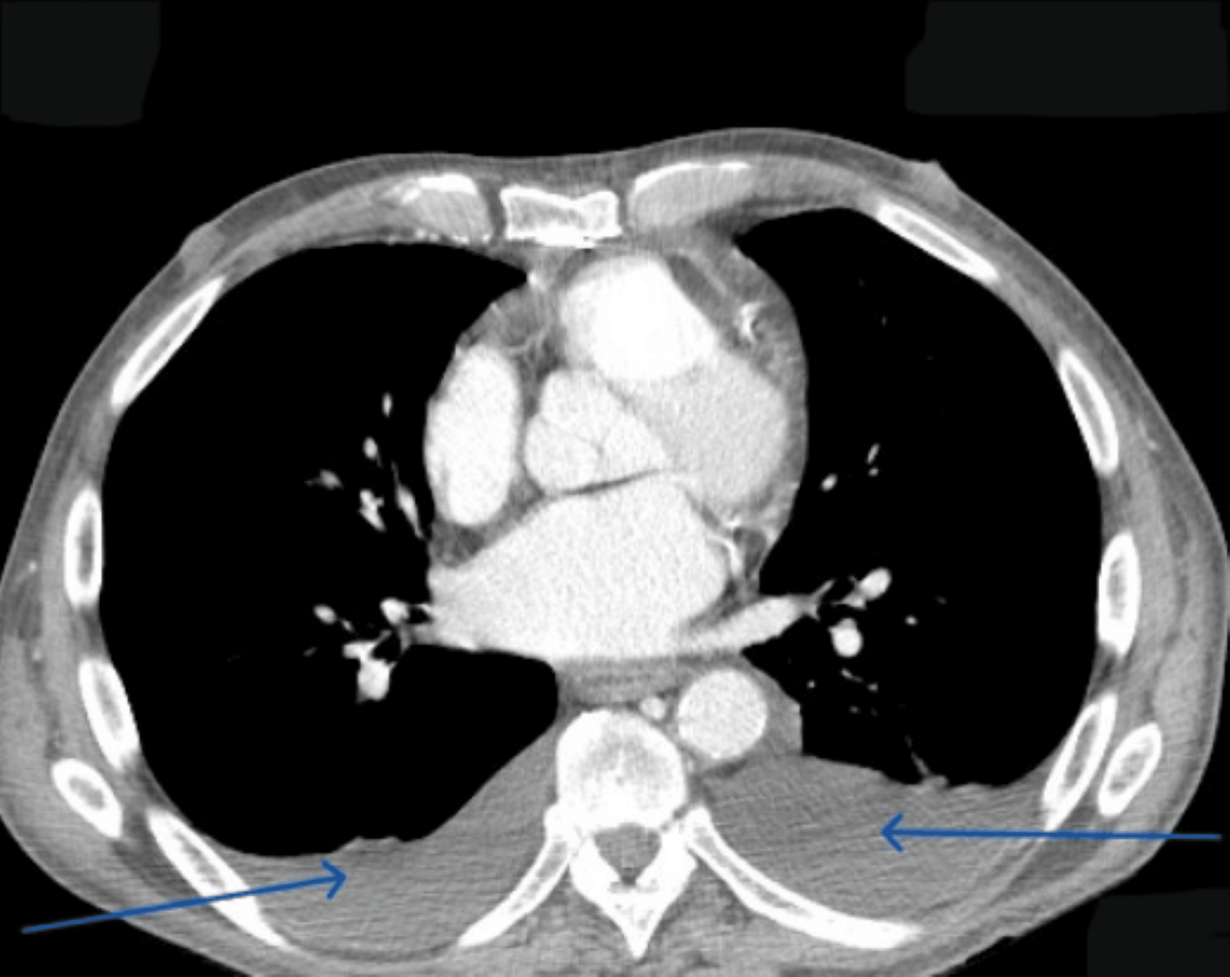
CT chest with bilateral pleural effusion (blue arrows).

Computed tomography (CT) chest revealed normal lung parenchyma, bilateral pleural effusion, and thickening of the pericardium (Figure 1).

**Figure 2:**
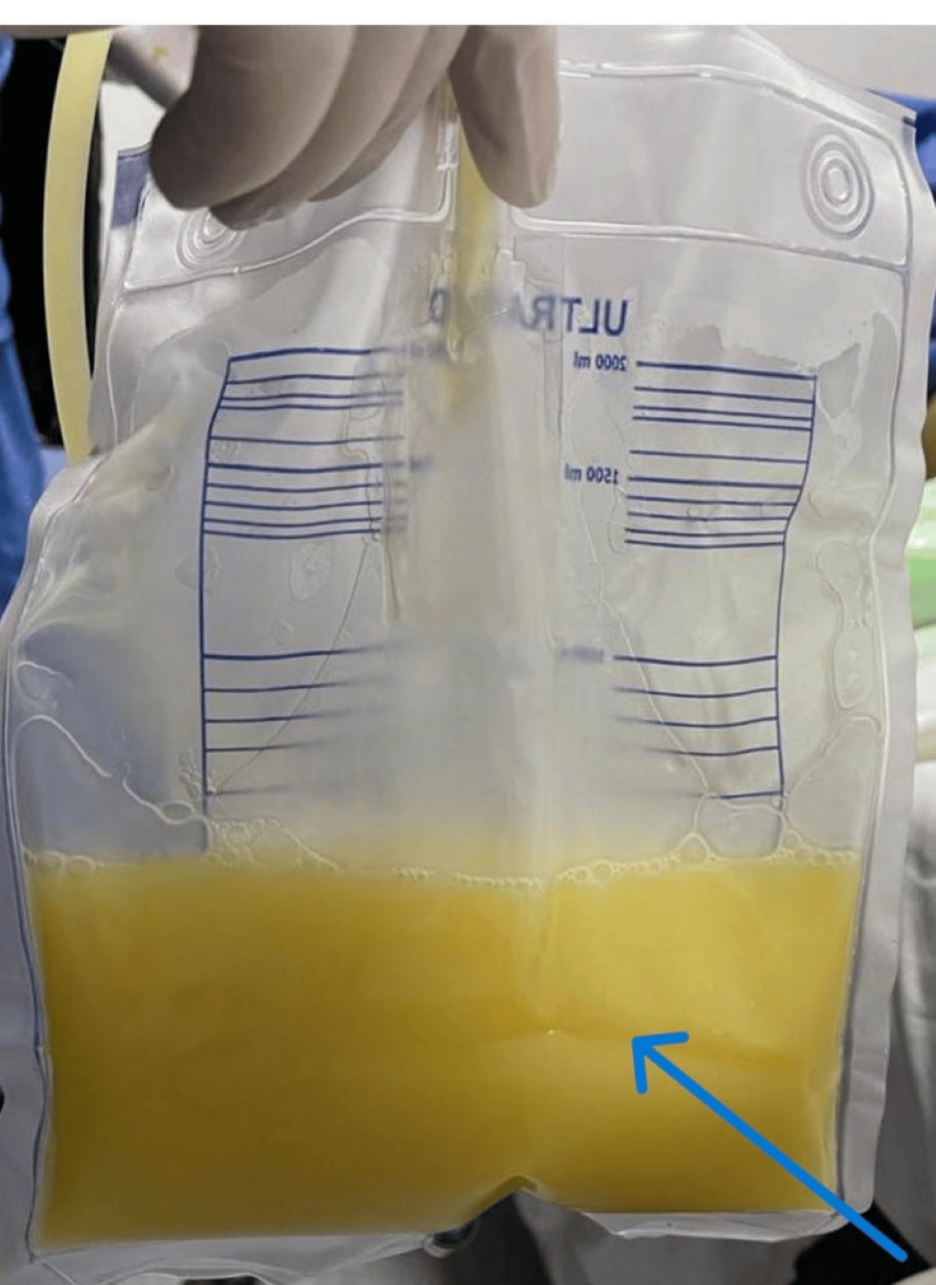
Post-drainage of pleural effusion (blue arrow).

Pleural fluid analysis was exudative in nature, normal triglyceride level, negative for microbiology, tuberculosis, and malignancy (Figure 2).

**Figure 3:**
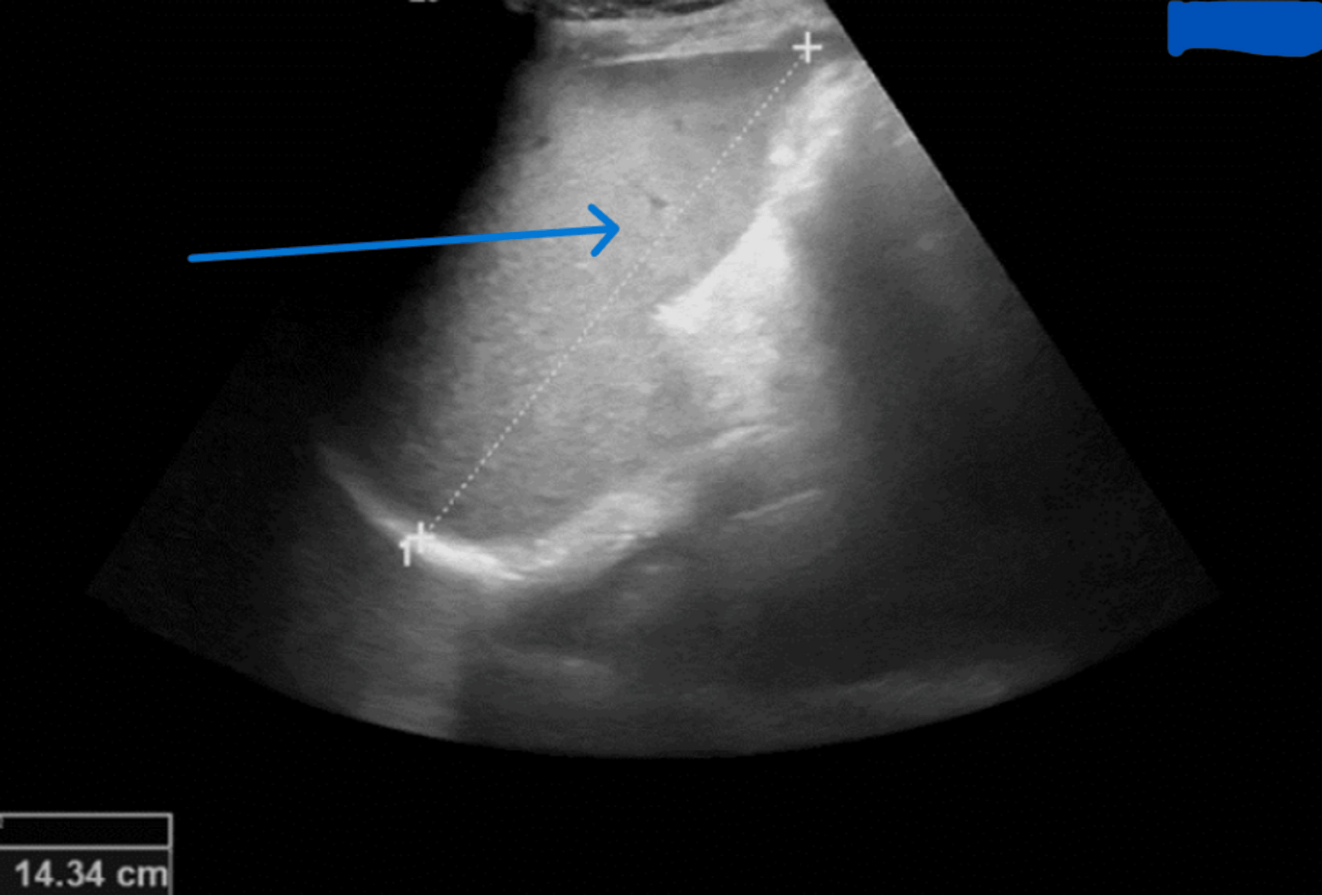
Ultrasound scan showing splenomegaly (blue arrow).

Echocardiography was done for suspected infective endocarditis but revealed normal ejection fraction with no detected abnormal findings. Ultrasound abdomen revealed mild splenomegaly (Figure 3).

**Figure 4:**
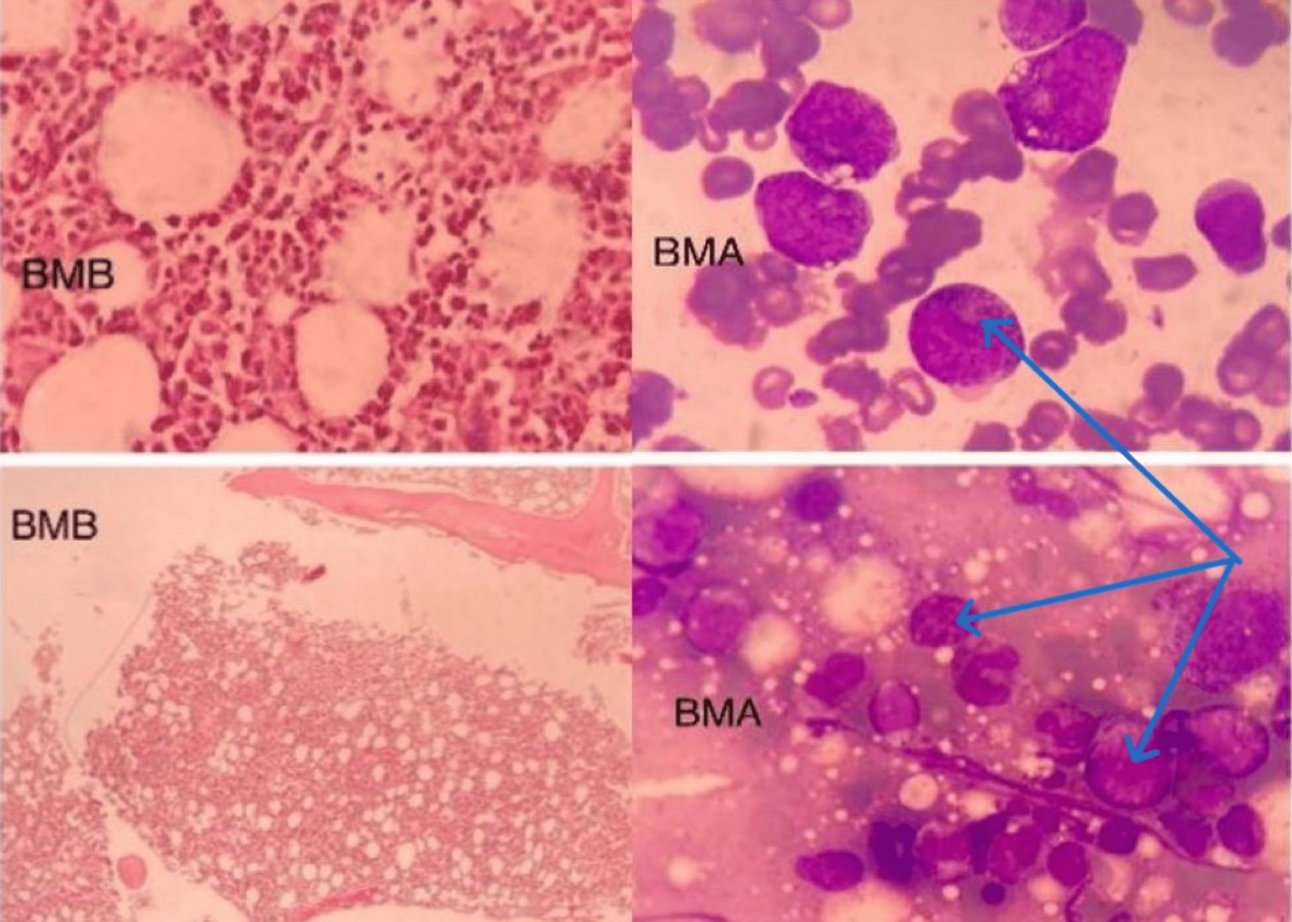
Bone marrow aspirate (BMA) and biopsy (BMB) showing hypercellularity (blue arrows) with no evidence of malignancy or infection.

Positron emission tomography (PET) CT scan showed no evidence of malignancy or septic focus. No suspicious findings in upper gastrointestinal endoscopy and colonoscopy. A bone marrow aspirate and biopsy revealed hypercellular bone marrow with a shift to the left, with no evidence of infection or malignant cells (Figure 4).

A multi-departmental team (MDT) comprising pulmonologists, hematologists, infectious disease experts, clinical pathologists, and rheumatologists received the case referral. Here, the Yamaguchi criteria were highlighted, and this case revealed a high diagnostic score for AOSD (Table 2) [4].

**Table 2:** Yamaguchi criteria

At least 5 criteria, including 2 major criteria and no exclusion criteria are required for diagnosis	
Major Criteria:	1- Fever ≥ 39 ⁰C lasting ≥ 1 week.
	2- Arthralgia lasting ≥ 2 week.
	3- Typical skin rash: maculopapular, non-pruritic, salmon-pink rash concomitant with fever spikes.
	4- Leucocytosis ≥ 10,000 cells/mm^3 ^with neutrophil polymorph-nuclear count ≥ 80%.
Minor Criteria:	1 - Pharyngitis or sore throat.
	2- Lymphadenopathy and/or splenomegaly.
	3 - Abnormalities in liver enzyme levels (aminotransferases).
	4 - Negative for rheumatoid factor or antinuclear antibodies.
Exclusion Criteria:	1 - Infection specially sepsis and Epstein-Barr viral infection.
	2- Malignant disease especially lymphoma.
	3- Inflammatory disease, especially polyarteritis nodosa.

Patient was managed initially with 500 mg of methylprednisolone for three consecutive days followed by 60 mg of prednisolone (1 mg/kg/day) in divided doses daily. He remained largely clinically stable, but fever and inflammatory markers remained elevated. It was here that tocilizumab (TCZ) was considered initially with a dose of 4 mg/kg with close monitoring of clinical and blood test response every four weeks for three months. With this addition, we saw a resolution in febrile episodes and noted down-trending of inflammatory markers for the first three weeks with a relapse of fever and elevated inflammatory markers a few days before the next dose of tocilizumab. This raised a concern that the disease is not optimally controlled (may need a higher or another pulse steroid dose, increasing the dose of tocilizumab to 8 mg/kg, initiation of disease-modifying antirheumatic drugs (DMARD) like ciclosporin, or switching to alternative biological treatment options like anakinra).

Ciclosporin was initiated as adjunctive therapy (third line with steroid and TCZ) for three months together with the same dose of tocilizumab (4 mg/kg). However, after renal function tests showed an elevated serum creatinine of 1.8 mg/dl, ciclosporin was stopped. Tocilizumab (in addition to prednisolone) was continued at the same dose (4 mg/kg) for another three months, but administered every three weeks instead of four weeks to better control the patient's symptoms. This approach resulted in an adequate clinical and biochemical response during that period. Despite this, a disease flare-up occurred, accompanied by a deterioration in the patient's general condition and an interleukin-6 (IL-6) level exceeding 5000 pg/ml. Immediate intervention with pulse steroids (methylprednisolone 1 gm for three consecutive doses) was administered, followed by an increased dose of tocilizumab at 8 mg/kg, which successfully achieved adequate disease control both clinically and biochemically.

At that point, a multidisciplinary team (MDT) meeting was conducted, and it was decided to continue lifelong tocilizumab administration every four weeks, along with a tapering steroid dose. During regular follow-up appointments, the patient maintained clinical and biochemical response with the same dose of tocilizumab (8 mg/kg) and 10 mg of daily prednisolone.

## Discussion

Usually, the onset of AOSD is in early adulthood, between 16 and 35 years old. People are mostly likely to contract AOSD, but in our reported case the onset was at an advanced age. Elderly-onset Still’s disease (EOSD) is reported in some cases, commonly in Japan, the USA, and Europe. One of the most used criteria for diagnosing EOSD is the Yamaguchi criteria. In elderly patients, a more severe course of the disease and more complications may be expected than in the younger group of patients with Still’s disease [5]. 

The pathogenesis is not entirely clear. There is no proof of infectious etiology; however, genetic variables and a range of viral triggers have been proposed as significant determinants. Whether the same pathogenic variables affect every patient is unknown. The most commonly recognized process is auto-inflammation, which results from the immune system being stimulated by one or more triggers, which then releases pro-inflammatory cytokines that cause systemic inflammation [6].

It is commonly known that macrophage activation plays a crucial role in triggering T helper 1 (Th1) cell cytokine activity. Pro-inflammatory cytokines, including interleukin (IL)-1, IL-6, and IL-18, are essential for the development of disease progression and stimulate ferritin production. As an acute-phase reactant, ferritin not only reflects the inflammatory state but also disrupts normal iron homeostasis, limiting free iron availability and potentially exacerbating oxidative stress.

The presence of AOSD has been suggested by a threshold for serum ferritin levels of 1000 ng/mL, which is five times the upper limits of normal (40-200 ng/mL). Extreme levels as high as 250,000 ng/mL have been documented, while extremely high values between 4000 and 30,000 ng/mL are not unusual. Generally speaking, people with AOSD have greater ferritin levels than patients with other autoimmune or inflammatory disorders. Whether ferritin contributes to the development of the disease or is merely an acute-phase reactant is yet unknown [7]. In our instance, ferritin was just slightly raised in spite of a significant inflammatory reaction and acute phase reactant.

Treatment of the systemic and arthritic aspects of AOSD with tocilizumab (TCZ), a humanized monoclonal antibody targeting the IL-6 receptor, has been demonstrated to be efficacious. Tocilizumab has been suggested as being preferable to IL-1 blocking medications in patients with predominant arthritic symptoms since high levels of IL-6 have been linked to an arthritic presentation in both systemic juvenile idiopathic arthritis (sJIA) and AOSD. According to a study by de Voysson et al., 27/28 patients (96%) and 30 out of 35 patients (80%) who received TCZ saw a rapid improvement in their systemic symptoms and articular presentation. Twenty percent of the patients were able to stop their steroid treatment, and over 80% of the patients were able to reduce their dosage [8].

Another study by Yuko Kaneko et al. demonstrated that inhibiting interleukin-6 (IL-6) with tocilizumab is effective for patients with adult-onset Still’s disease (AOSD) refractory to glucocorticoid therapy [9]. Tocilizumab significantly enabled a reduction in glucocorticoid dosage, which is crucial given the adverse effects associated with long-term glucocorticoid use. The study confirmed tocilizumab's acceptable safety profile, highlighting its potential as a viable treatment option that can improve the quality of life for AOSD patients by effectively managing symptoms and minimizing glucocorticoid-related side effects [9].

Patient quality of life has improved dramatically for those with AOSD as a result of recent advancements and the introduction of biologic medications. IL-1 and IL-6 suppression is the most effective long-term treatment approach, according to safety and efficacy evidence [10].

While a randomized controlled trial has not yet assessed the safety and effectiveness of TCZ for AOSD, a number of case and pilot studies have demonstrated that TCZ treatment improved the clinical symptoms and signs of AOSD in patients who had not responded to standard treatment plans or biologics, such as anakinra and TNF inhibitors. These results suggest that TCZ might replace other biologics as the first-line treatment for AOSD [11].

## Conclusions

This case report highlights the challenges of managing an older patient with adult-onset Still's disease (AOSD) presenting with persistent fever, elevated acute phase reactants, and pleural effusion despite high-dose steroid therapy. Treating AOSD in older adults is complicated by age-related factors like reduced organ function and comorbidities, increasing sensitivity to medications. Tocilizumab, an IL-6 receptor antagonist, was considered to address the cytokine storm but required careful balancing due to the increased risk of infection from immunosuppression. Continuous reassessment of potential infectious, autoimmune, and malignant causes was crucial. The case underscores the importance of close monitoring and interdisciplinary collaboration, emphasizing the need for tailored treatment approaches that consider the patient’s age, comorbidities, and evolving clinical response.
